# Cerebral blood flow during cardiopulmonary bypass in pediatric cardiac surgery: the role of transcranial Doppler – a systematic review of the literature

**DOI:** 10.1186/1476-7120-4-47

**Published:** 2006-12-13

**Authors:** Angelo Polito, Zaccaria Ricci, Luca Di Chiara, Chiara Giorni, Claudia Iacoella, Stephen P Sanders, Sergio Picardo

**Affiliations:** 1 Department of Pediatric Cardiology and Cardiac Surgery, Bambino Gesù Hospital, Rome, Italy

## Abstract

**Background:**

Transcranial Doppler Ultrasound (TCD) is a sensitive, real time tool for monitoring cerebral blood flow velocity (CBFV). This technique is fast, accurate, reproducible and noninvasive. In the setting of congenital heart surgery, TCD finds application in the evaluation of cerebral blood flow variations during cardiopulmonary bypass (CPB).

**Methodology:**

We performed a search on human studies published on the MEDLINE using the keyword "trans cranial Doppler" crossed with "pediatric cardiac surgery" AND "cardio pulmonary by pass", OR deep hypothermic cardiac arrest", OR "neurological monitoring".

**Discussion:**

Current scientific evidence suggests a good correlation between changes in cbral blood flow and mean cerebral artery (MCA) blood flow velocity. The introduction of Doppler technology has allowed an accurate monitorization of cerebral blood flow (CBF) during circulatory arrest and low-flow CPB. TCD has also been utilized in detecting cerebral emboli, improper cannulation or cross clamping of aortic arch vessels. Limitations of TCD routine utilization are represented by the need of a learning curve and some experience by the operators, as well as the need of implementing CBF informations with, for example, data on brain tissue oxygen delivery and consumption.

**Conclusion:**

In this light, TCD plays an essential role in multimodal neurological monitorization during CPB (Near Infrared Spectroscopy, TCD, processed electro encephalography) that, according to recent studies, can help to significantly improve neurological outcome after cardiac surgery in neonates and pediatric patients.

## Background

Transcranial Doppler Ultrasound (TCD) is currently used as a sensitive, real time tool for monitorization of cerebral blood flow velocity (CBFV). From the first clinical application by Aaslid in 1982 [1], TCD has been extensively used in clinical routine, in particular during neurosurgery and vascular surgery. In the setting of congenital heart surgery, TCD finds application in the evaluation of cerebral blood flow variations as well as the presence of emboli during, before and after cardiopulmonary bypass (CPB). The present review aims to describe technical characteristics and clinical applications of TCD in pediatric cardiac surgery with a critical discussion of most important literature published in this field.

### Theoretic principles

The available instruments emit pulsed-wave ultrasounds at 2–4 MHz who penetrate and scatter the tissue. The ultrasonic waves are then backscattered from moving red blood cells with a shifted frequency toward the receiver, placed in the same doppler probe of the transmitter. The frequency shift lies in the audible range. The frequency (Doppler) shift can be expressed as follows:

F = 2 * Fo * v * cos∝/c

where F is Doppler shift (Hz), Fo = mean frequency of the emitted ultrasound, v = blood flow velocity (cm/s), ∝ = angle between the direction of the transmitted sound beam and the axis of the blood flow and c = velocity of sound in tissue. Thus, F is proportional to blood flow velocity (v) if the angle and the frequency of emitted ultrasound remains constant. The formula also shows that the angle between the beam's direction and blood should be kept close to 0 in order to minimize the possibility of measuring errors (the maximum value of F can be found at ∝ = 0).

### Technical considerations

**Figure 1:**
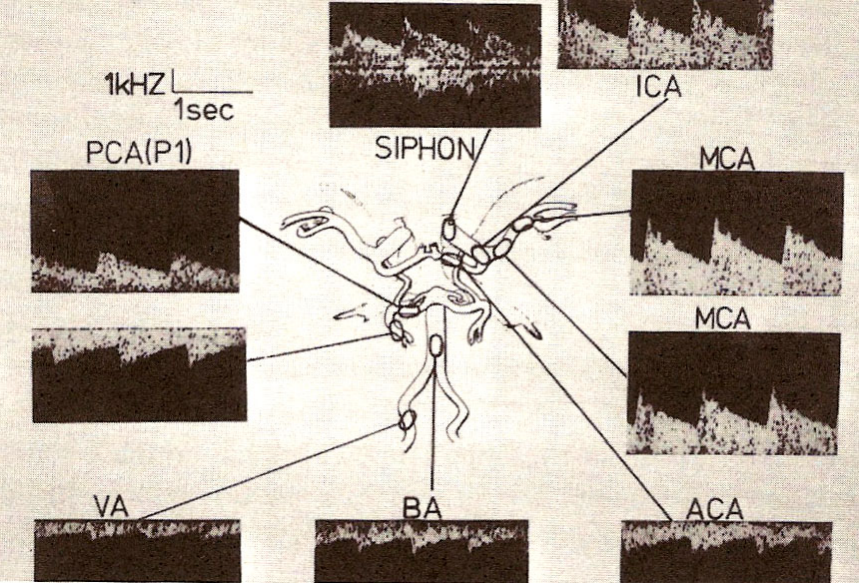
Example of wave's form obtained with TCD (From "Argomenti di Neurosonologia ed Emodinamica Cerebrale", M. Visocchi, Ed. Avenue Media, Bologna).

Several positions can be used to examine the basal cerebral arteries (Figure 1), but the most reproducible and comfortable tecnique for clinical use in pediatric patients is to insonate the middle cerebral artery (MCA) through the temporal window which can be found about 1 cm in front of the external auditory meatus and roughly 1–2 cm above the zygomatic arch. The ultrasonic beam is directed horizontally. The depth of the sample volume and angle of insonation is adjusted until the bifurcation of the MCA and the anterior cerebral artery (ACA) is found (positive deflection toward the transducer from the MCA and a retrograde signal with a negative deflection from the ACA). Normally the clinician should find a stable baseline at the beginning of evaluation and compare it with further values (Table 1).

**Table 1:** Normal transcranial doppler velocities in the middle cerebral arteries obtained through the temporal window in awake children without cardiovascular disease, expressed as mean +/- SD. (From 31).

Age	Depth(mm)	Mean velocity (cm/s)	Peak systolic velocity (cm/s)	End-diastolic velocity (cm/s)
0–3 mo	25	24–42+/-10	46–75+/-15	12–24+/-8
3–12 mo	30	74 +/- 14	114 +/- 20	46+/- 9
1–3 yr	35–45	85 +/- 10	124 +/- 10	65 +/- 11
3–6 yr	40–45	94 +/- 10	147 +/- 17	65 +/- 9
6–10 yr	45–50	97 +/- 9	143 +/- 13	72 +/- 9
10–18 yr	45–50	81 +/- 11	129 +/- 17	60 +/- 8

### Relation between blood flow velocity and cerebral blood flow

According to the Hagen-Poiseuille law, the flow in a rigid tube is

F = P * π * r^4^/8 * η * l

Where r is the radius of the tube, l is the length of the tube, P is the difference between the pressure at the beginning and at the end of the tube, η is the viscosity of the fluid.

The relation between the flow and the velocity (v) is

F = v * π * r^2^

From the previous formula and from the Hagen-Poiseuille law we obtain

v = P * r^2^/8 * η * l

The previous formulas allow us to conclude that the flow (ml/min) in a vessel is dependent on the radius of the vessel and on the flow velocity within the vessel. The velocity is dependent on viscosity (hematocrit) of the blood, on the radius of the vessel and on the perfusion pressure in the vessel. Assuming that the MCA diameter remains unchanged, any changes in velocity would reflect changes in flow. Several experimental works have provided evidences that the diameter of the MCA does not change significantly during cardiac operations, the vasomotor action being confined to resistance arteries and arterioles, as demonstrated by previous works [2,3].

## Methodology

We performed a search on MEDLINE using the keyword "trans cranial Doppler" crossed with "pediatric cardiac surgery" AND "cardio pulmonary by pass", OR deep hypothermic cardiac arrest", OR "neurological monitoring". Our search was limited to human studies published up to December 2005. After abstract collection, a consensus decision by all authors was made in order to select eligible full text articles. Reasons for exclusion were: age groups (adults), abstracts with unavailable full text, and language other than English.

34 articles were selected. Of these, 23 have been taken into consideration for discussion [4-20,22-27]. 9 were excluded because age group was uncorrect, full text was not available or absent, language was different from English or consensus among researchers about paper quality was not reached. In one case [31] a book chapter was selected in order to present normal transcranial doppler velocities in the middle cerebral arteries in awake children (table 1).

## Literature analysis

### TCD and cerebral blood flow modifications (table 2)

**Table 2:** TCD and cerebral blood flow modifications. MCA: middle cerebral artery. CPB: cardiopulmonary bypass. CBF: cerebral blood flow.

**Author [reference]**	**Journal (Year of Publication)**	**Type of study and Number of patients**	**Main findings**
Lindegaard KF [4]	Stroke (1987)	Observational on 7 adult patients	Linear relationship between flow volume and blood velocity in MCA is present.
Weyland A [5]	Anesthesiology (1994)	Observational on 15 adult patients	Linear relationship between flow volume and blood velocity in MCA is present before and after CPB but cannot reliably predict percentage changes in CBF during CPB.
Bishop CCR [6]	Stroke (1986)	Observational on 17 adult patients	Changes in MCA velocity reliably correlate with changes with CBF evaluated with Xenon^133^
Rosemberg A [7]	Pediatric Research (1985)	Experimental in newborn lambs	Changes in cerebral blood flow velocity are useful qualitative measures of changes in cerebral blood flow
Trivedi U [8]	Annals of Thoracic Surgery (1997)	Randomized trial on 60 adult patients receiving either α-stat or pH stat management based CPB	Measurement of MCA velocity by TCD expressed as relative changes of a pre-CPB level can be used to examine CBF changes during CPB

**Figure 2:**
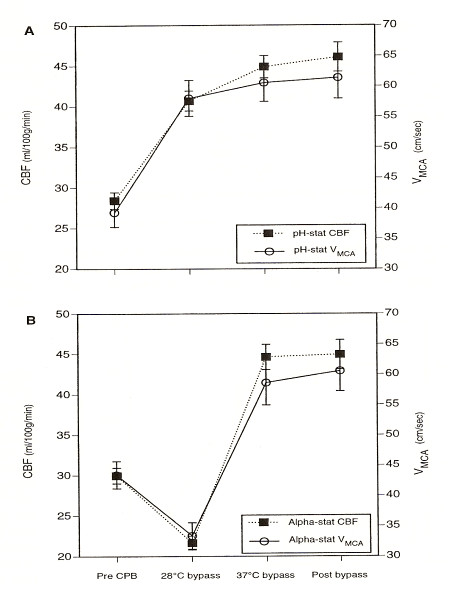
Changes in cerebral blood flow (CBF) and cerebral blood flow velocity (CBFV) in patients subjected to (A) pH-stat management and (B) α-stat acid-base management (From 8).

Many studies have used TCD in order to evaluate changes of cerebral perfusion measuring blood flow velocities [4,5]. As the diameter of the insonated vessel is not know in any individual, it is not possible to evaluate the absolute value of cerebral blood flow. However, the variation in cerebral blood flow (CBF) is determinant, rather than the absolute value. The reliability of correlation between changes in MCA velocity and CBF (measured using intravenous Xenon-133) has been first validated by Bishop and coworkers [6] in an experimental work that compared the MCA flow velocities and CBF in symptomatic patients with cerebrovascular disease in normo and hypercapnic conditions. These findings have been confirmed in an experimental work by Rosemberg [7], who demonstrated that changes in cerebral blood flow velocity are useful qualitative measures of changes in cerebral blood flow in paralyzed newborn lambs. Trivedi et al. [8] showed marked similarities between changes in the velocity in the MCA and changes in the estimated hemispheric CBF measured with Xenon-133 clearance technique (Figure 2).

### Role of TCD during deep hypothermic cardio pulmonary bypass, deep hypothermic circulatory arrest and low flow cardio pulmonary bypass (table 3)

**Table 3:** Role of TCD During Deep hypothermic Cardio Pulmonary Bypass (DHCPB), Deep Hypothermic Circulatory Arrest (DHCA) and Low Flow Cardio Pulmonary Bypass (LFCPB). MCA: middle cerebral artery. CPB: cardiopulmonary bypass. CBF: cerebral blood flow. CMRO_2_: cerebral metabolic rate of oxygen, CPP: cerebral perfusion pressure.

**Author [reference]**	**Journal (Year of Publication)**	**Type of study and Number of patients**	**Main findings**
Norwood WI [9]	Journal of Thoracic Cardiovascular Surgery (1979)	Experimental study on neonatal rats	Hypothermia during CPB reduces metabolic activity, CBF and CMRO_2_, so that it is possible to maintain energy stores and provide organ protection during low flow state
Greeley WJ [10]	Journal of Thoracic Cardiovascular Surgery (1991)	Prospective study on 46 pediatric patients	DHCA changes cerebral metabolism and blood flow after the arrest period
Fox LS [11]	Journal of Thoracic Cardiovascular Surgery (1984)	Experimental study on 9 monkeys randomly assigned to 4 perfusion flow rates varying from 0.25 to 1.75 L/min/m^2^	All areas of the brain remain perfused, even at low perfusion flow rates, during profoundly hypothermic cardiopulmonary bypass, and brain oxygen consumption is maintained in part by increased oxygen extraction and in part by redistribution of the perfusate from the remaining body to the brain
Rebeyka IM [12]	Annals of Thoracic Surgery (1987)	Experimental study on 6 dogs and prospective study on 5 patients subjected to brief periods of low-flow CPB (Q = 1.0 L/min/m2.) at 21 degrees to 25 degrees C°.	In the absence of cerebral vascular disease, the flow rate threshold for incurring functional cerebral injury during hypothermic (25 degrees C) nonpulsatile CPB is less than 1.0 L/min/m^2^.
Taylor R [13]	Anesthesia Analgesia (1992)	Observational study on 25 infants and neonates	1) Autoregulation is preserved during normothermic CPB, it begins to be altered at temperature less than 25°C, and it is lost at temperature less than 20°C. 2) A significant decrease in CPP and CBF is shown during extreme low flow CPB.
Jonassen A [14]	Journal of Thoracic Cardiovascular Surgery (1995)	Observational on 37 pediatric patients	Detectable cerebral blood flow at pump flow rate and mean arterial pressure values of 27 mmHg (lower than those reported by Taylor).
Greeley WJ [15]	Circulation (1989)	Observational on 67 pediatric patients	During CPB rewarming, CBF returns to baseline values, except in patients exposed to periods of DHCA where CBF remains decreased.
Astudillo R [16]	Annals of Thoraci Surgery (1993)	Observational on 22 small children	Low cerebral perfusion immediately following DHCA is characterized by a prolonged period of absent diastolic CBFV in MCA while patients subjected to continuous low-flow perfusion technique showed a CBFV close to baseline values at skin closure.
Rodriguez R [17]	Journal of Thoracic Cardiovascular Surgery (1995)	Randomized trial on 16 infants treated with or without 10 minutes of cold reperfusion before rewarming after DHCA.	A delay in rewarming on reperfusion after DHCA may be beneficial as demonstrated by recovery of a diastolic doppler signal.
Zimmerman A [18]	Journal of Thoracic Cardiovascular Surgery (1997)	Observational on 28 neonates	Cerebral perfusion can be detected by TCD in the MCA in some neonates at bypass flow as low as 10 ml/kg per minute.

**Figure 3:**
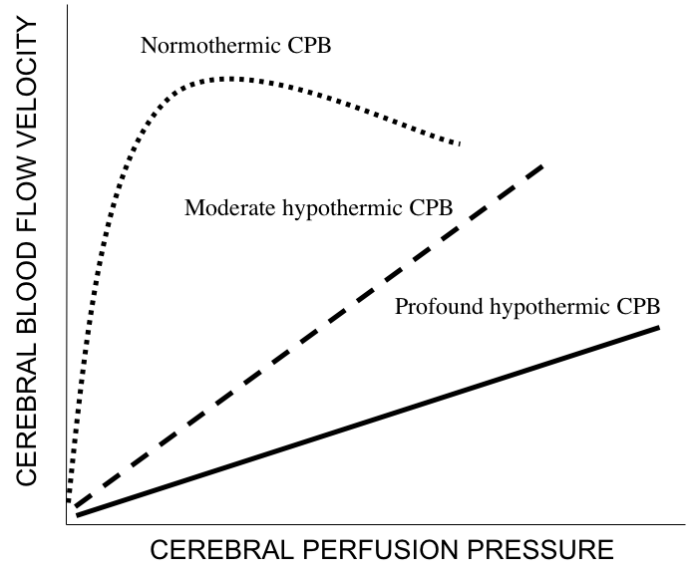
Schematic representation of the relationship of cerebral blood flow velocity (CBFV) and cerebral perfusion pressure (CPP) during normothermic (dotted line), moderate hypothermic (dashed line) and profound hypothermic cardiopulmonary bypass (CPB) (solid line) (adapted From 13).

**Figure 4:**
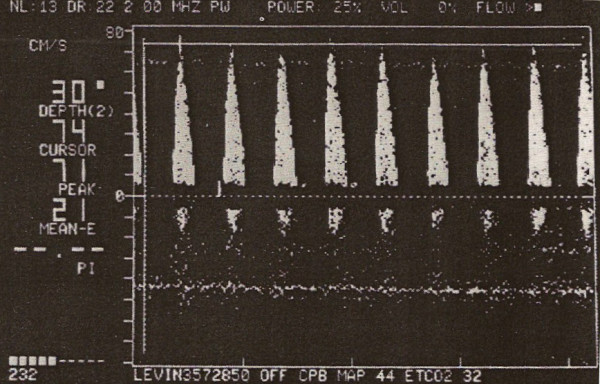
Absence of diastolic forward flow velocity after cardiopulmonary bypass with profound hypothermia (From 14).

**Figure 5:**
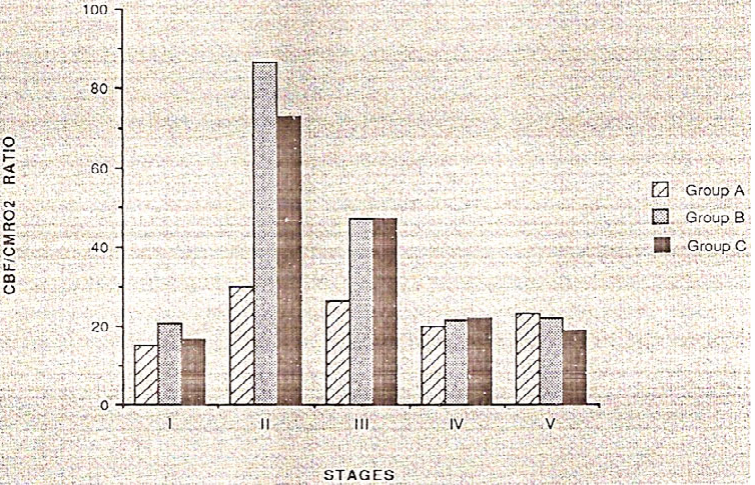
Cerebral blood flow/cerebral metabolic rate of oxygen coupling (CBF/CMRO_2_) during cardiac surgery. Group A-moderate hypothermia-, Group B-deep hypothermic cardiopulmonary bypass (CPB) with maintenance of continuous flow, Group C-deep hypothermic circulatory arrest. Stage I = pre CPB; II and III = CPB cold; IV = rewarm; V = post CPB (From 15).

**Figure 6:**
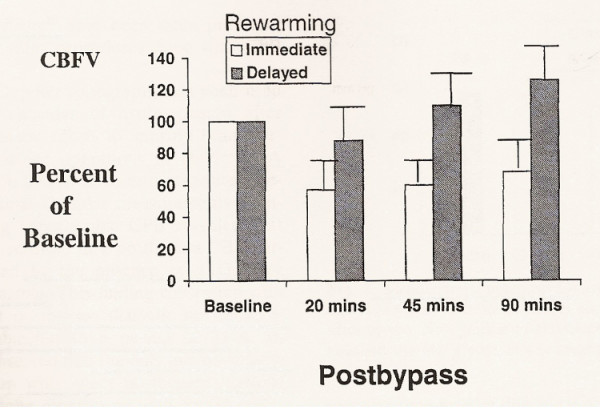
Mean cerebral blood flow velocities (CBFV) expressed in percentages of baseline for patients with immediate and delayed rewarming. Flow velocities remained below the baseline in immediate rewarming group, but for group of delayed rewarming mean flow velocity was comparable with baseline at all postbypass measurement (From 17).

The introduction of deep hypotermic cardiopulmonary bypass (DHCPB) with or without deep hypothermic circulatory arrest (DHCA) in children who need complex aortic arch reconstruction has substantially improved operating conditions and therefore reduced cardiac morbidity. The aim of hypothermia during CPB is to reduce metabolic activity, CBF and cerebral metabolic rate of oxygen (CMRO_2_), so that it is possible to maintain energy stores and provide organ protection during low flow state [9,10]. Profound hypothermia with continuous low-flow cardiopulmonary bypass (low-flow CPB) has been suggested as being superior to DHCA in preventing neurological damage [11,12], hypothetically providing an indefinite period of cerebral perfusion. The most important factor ruling cerebral hemodynamics is cerebral autoregulation, in order to maintain CBF constant throughout a wide range of arterial pressure. This autoregolatory mechanism is deeply affected by temperature. Taylor and coworkers [13] found that autoregulation is preserved during normothermic CPB, it begins to be altered at temperature less than 25°C, and it is lost at temperature less than 20°C, while previous studies had shown that autoregulation is intact during moderately hypothermic CPB (25° to 32°C) [10], and it is lost during deep hypothermic CPB (18° to 22°C) (Figure 3). However, this loss of autoregulation is most likely caused by a state of "cold-induced vasoparesis", in which cerebral vascular resistance increases with temperature reduction. Jonassen and coworkers [14] showed that a significant proportion of patients treated with profound hypothermia and either low-flow CPB or circulatory arrest exhibited a TCD pattern consistent with increased cerebral vascular resistance in the early postoperative period, whereas this pattern was not present in patients treated with moderately hypothermic CPB. There was a tendency for this pattern to occur with greater frequency in patients who had a period of circulatory arrest (Figure 4). While CBF decreases in a linear manner, CMRO_2 _decreases exponentially with temperature reduction. Therefore, CBF/CMRO_2 _during DHCPB increases favouring luxury perfusion of the brain. Normal coupling of CBF/CMRO_2 _is present before and after CPB, as well as during normothermic CPB and α-stat management [10]. During CPB rewarming, CBF returns to baseline values, except in patients exposed to periods of DHCA where CBF remains decreased (Figure 5) [15]. Astudillo et al. [16] demonstrated that low cerebral perfusion immediately following DHCA is characterized by a prolonged period of absent diastolic CBFV in MCA: this finding was explained by an increased intracranial pressure after total circulatory arrest procedure, while patients subjected to continuous low-flow perfusion technique showed a CBFV close to baseline values at skin closure. It must be remarked however, that a significant age difference between the two groups was present, being the patients subjected to circulatory arrest younger than the patients in the non arrest group. An interesting finding from the group of Rodriguez [17] is that a delay in rewarming on reperfusion after DHCA improved recovery of a diastolic doppler signal compared with patients who underwent immediate rewarming. In the group undergoing cold reperfusion, postbypass CBF velocity was not different from baseline (Figure 6). Taylor and coworkers [13] used the TCD as an indicator of perfusion during repair of congenital heart defects requiring moderate or profound hypothermia and low-flow CPB. The principal finding of this study was the immediate loss of detectable CBFV in the middle cerebral artery when cerebral perfusion pressure (CPP) decreased below 9 mmHg: they clearly conclude that CPP is a crucial parameter, rather then pump flow rate, in impacting brain perfusion. They also confirmed the loss of cerebral autoregulation between 23° and 25°. However, Jonassen et al [14], in agreement with data from van der Linden [2], showed detectable cerebral blood flow at pump flow rate and mean arterial pressure (MAP) values lower than those reported by Taylor. Possible explanations may include the use of vasodilators, the increased sensitivity of the TCD apparatus by removal of the low-pass filter and the avoidance of jugular central venous lines that can theoretically impede regional cerebral venous drainage in small infants and thus decreasing CPP. A more recent study from Zimmermann et al. [18] performed in 28 neonates undergoing the arterial switch operation with α-stat acid-base management, showed that cerebral perfusion can be detected by TCD in the MCA in some neonates at bypass flow as low as 10 ml/kg per minute. However a minimum bypass flow rate of 30 ml/kg per minute was needed to detect cerebral perfusion in all neonates. All patients with a MAP of 19 mmHg or greater, regardless of pump flow rate, had detectable cerebral perfusion by TCD but correlation between MAP and CPB pump flow rates was minimal, confirming the conclusions of Taylor that mean arterial blood pressure alone is a poor indicator of CPP.

### Role of TCD during regional low-flow perfusion for neonatal aortic arch reconstruction (table 4)

**Table 4:** Role of TCD during regional low-flow perfusion for neonatal aortic arch reconstruction.

**Author [reference]**	**Journal (Year of Publication)**	**Type of study and Number of patients**	**Main findings**
Pigula F [19]	Journal of Thoracic Cardiovascular Surgery (2000)	Observational on 6 neonates	Significant decreases are shown in both cerebral blood volume and oxygen saturation in children who underwent repair with DHCA as compared with children with RLFP.
Andropulos D [20]	Journal of Thoracic Cardiovascular Surgery (2002)	Observational on 34 neonates	The use of TCD is able to maintain cerebral oxygen saturations and blood flow velocities within 10% of baseline, in order to prevent cerebral hyperperfusion during periods with high NIRS saturation values.

The aim of regional low flow perfusion (RLFP) technique is to minimize the effect of DHCA on the brain maintaining cerebral blood volume and oxygen saturation while providing the same surgical exposure obtained with DHCA alone. Using near infrared spectroscopy (NIRS) technology Pigula et al. [19] documented significant decreases in both cerebral blood volume and oxygen saturation in children who underwent repair with DHCA as compared with children with RLFP. When the use of RLFP rate is guided by NIRS, cerebral oxygen saturation was the same of that provided by standard CPB support. Andropoulos and coworkers [20] found that the use of TCD ultrasonography might add further informations during bypass and RLFP. In particular, since NIRS monitor is not able to display oxygen saturation higher than 95%, it only detects inadequate blood flow (desaturation) but not excessive CBF (hypersaturation). The potential dangers of excessive CBF include cerebral edema and intracranial hemorrage. The use of TCD is thus of great importance in order to maintain cerebral oxygen saturations and blood flow velocities within 10% of baseline, in order to prevent cerebral hyperperfusion during periods with high NIRS saturation values. Noteworthy, a significant difference in the mean bypass flow rate necessary to maintain the baseline oxygen saturation and CBFV was present between the two studies. Reacquisition of baseline cerebral blood volume and cerebral oxygen saturations were accomplished with a RLFP of 20 ml/Kg/min and 63 ml/Kg/min by Pigula and Andropoulos, respectively. A possible explanation of this significantly different perfusion management could be attributed to the fact that Andropoulos and coworkers obtained a higher cerebral vasodilation due to a different acid-base management technique (Ph-stat versus α-stat used by Pigula) and to the use of phenoxybenzamine or phentolamine hydrocloride during CPB.

### Alpha versus Ph stat blood gas management (table 5)

**Table 5:** Miscellaneous. CBFV: cerebral blood flow velocity. DHCPB: deep hypothermic cardiopulmonary bypass.

**Author [reference]**	**Journal (Year of Publication)**	**Type of study and Number of patients**	**Main findings**
Trivedi U [8]	Annals of Thoracic Surgery (1997)	Randomized trial on 60 adult patients receiving either α-stat or pH stat management based CPB	A decrease of CBF (evaluated by Xenon-133 and CBFV) is shown during 28° bypass in patients subjected to α-stat management. This is associated with a significant reduction in PaCO_2_, while there is no reduction in patients subjected to pH-stat management.
Patel RL [22]	European Journal of Cardiothoracic Surgery (1993)	Randomized protocol on 70 adult patient undergoing α-stat or ph stat CPB	The cerebral extraction ratio for oxygen indicated a degree of mismatch of cerebral perfusion and demand during CPB in both pH-stat and alpha-stat groups. This mismatch was more pronounced in the pH-stat group than in the alpha-stat group, indicating greater disruption in cerebral autoregulation in the former group.
Gruber E [23]	Anesthesia Analgesia (1999)	Prospective randomized study on 35 neonates and infants managed with different hematocrit values	An inverse relation is present between hematocrit and CBFV during DHCPB
Rodriguez R [24]	Annals of Thoracic Surgery (2000)	Observational on 124 children	Aorto-venous can impair cerebral perfusion which may be effectively followed by Doppler flow.

It is well known that CO_2 _has a profound influence on CBF [21] with increasing CO_2 _resulting in an increase in CBF. During CPB, CBF increases with increasing arterial carbon dioxide tension, but this response is diminished by deep hypothermia and age less than 1 year. In the pH-stat management PaCO_2 _is maintained at 40 mmHg regardless of temperature changes (temperature-corrected), while α-stat management PaCO_2 _is not adjusted (temperature-uncorrected). Trivedi et al. [8] showed a decrease of CBF (evaluated by Xenon-133 and CBFV) during 28° bypass in patients subjected to α-stat management. This was associated with a significant reduction in PaCO_2_, while there was no reduction in patients subjected to pH-stat management (Figure 2). Other studies, however, performed by another group demonstrated that during α-stat management cerebral blood flow velocity measured by TCD was less pressure passive than during pH-stat management and that there was a better matching of cerebral metabolism to CBFV with α-stat management [22].

### Hematocrit (table 5)

There is an inverse relation between hematocrit and and CBFV during DHCPB in neonates and infants, although the optimal hematocrit to perform CPB has not yet been determined [23].

### Effects of cannulation for cardiopulmonary bypass (table 5)

**Figure 7:**
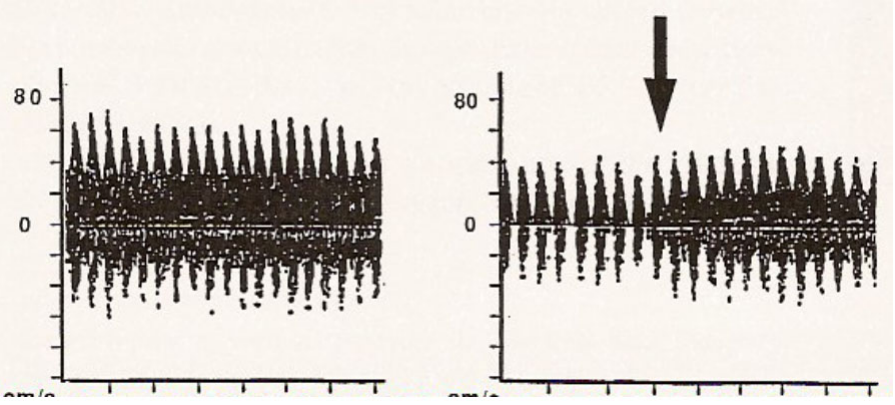
Transcranial doppler waveforms before and during superior vena caval obstruction and cannula repositioning (arrow) (From 24).

Rodriguez et al. [24] showed that aortic and venous cannulations for CPB in children can hesitate in transient reductions in CBFV and MAP and that usually these alterations are compensated within the next 40 seconds. Reported undesirable cerebral effects were more frequent in young infants with cannulation of right atrium. Superior Vena Cava obstruction during venous cannulation resulted in an increased pressure in the internal jugular vein and in the absence of diastolic Doppler flow (Figure 7).

## Clinical implications

### Multimodality neurological monitoring (table 6)

**Table 6:** Clinical implications.

**Author [reference]**	**Journal (Year of Publication)**	**Type of study and Number of patients**	**Main findings**
Austin E III [25]	Journal of Thoracic and Cardiovascular surgery	Observational on 250 pediatric patients.	Interventions based on neurophysiologic monitoring (NIRS, TCD, EEG) decrease postoperative neurologic sequelae and reduce hospital lenght of stay.
Andropoulos D [26]	Anesthesia Analgesia (2004)	Literature review and institutional data report	Multimodal neurological monitoring in conjunction with a treatment algorithm may improve neurological outcome.
O'Brien [27]	Anesthesiology (1997)	Observational on 25 children	Microemboli can be detected in the carotid arteries of children during repair of congenital heart disease and are especially prevalent immediately after release of the aortic cross clamp.

Recent studies showed the beneficial effects of simultaneous neurological monitoring (NIRS, TCD, processed electro encephalography). Austin et al. [25] showed the potential benefit of intraoperative interventions based on an intervention algorithm previously used in adult cardiac surgery and modified for pediatric use (Table 7). The algorithm design triggers specific interventions when specific clinical parameters change. Two groups of patients were randomized to the intervention or no intervention group in response to any detected perfusion abnormality. 70% of patients in this study experienced significant changes in one or more monitored variables. The incidence of neurological sequelae (i.e. seizure, movement, vision or speech disorders) between patients with no change of neurophysiologic values and patients who received interventions in response to a change in any of the monitored variables was the same (6–7%), while neurological damage was 26% among patients who did not receive any treatment. The proportion of neurological sequelae in no-interventions patients discharged from the hospital within 1 week (32%) was significantly lower than that observed in either the intervention (51%) or no change group (58%). The TCD was responsible for 37% of detected problems, while in another study from Andropoulos et al. [26] the TCD alone was responsible for only the 10% of interventions.

**Table 7:** Intervention algorithm initiated by EEG slowing and/or cerebral venous oxygen desaturation. /: no change; +: increase; -: decrease; TCD: trans cranial doppler; CPB: cardio pulmonary bypass (From 25).

**TEMPERATURE**	**BLOOD PRESSURE**	**TCD**	**NOTIFICATION**	**INTERVENTION**
*Prebypass*				
/	/	- peak velocity	Aorta obstructed	Adjust aortic cannula
/	/	- diastolic velocity	Cava obstructed	Asdjust venous cannula
*CPB*				
/	/	+ peak velocity	Hyperemia	- Pump flow
/	/	Gas emboli	Gas emboli	Deair, repair circuit
+	/	- peak velocity	Flow metabolism uncoupling	+BP; - metabolic demand
/	/	- peak velocity	Low cerebral flow	Adjust aortic cannula/clamp; + Pump flow
*Postbypass*				
/	/	- Diastolic velocity	Cerebral edema	Mannitol; ultrafiltration
*Anytime*				
/	-	- Peak velocity	Loss of autoregolation	+ BP; neuroprotection
+ *EEG frequency*				
/	/, +	/, + peak velocity	Insufficient sedation	+ Sedation

### Other clinical use of TCD in pediatric cardiosurgery: detection of emboli (table 6)

TCD can also easily detect and count cerebral emboli; they are described as high intensity transient signals (HITS). A study from O'Brien [27] showed that microemboli can be detected in the carotid arteries of children during repair of congenital heart disease and are especially prevalent immediately after release of the aortic cross clamp. However the TCD cannot distinguish between true emboli and false positive artefacts. The number of emboli detected during pediatric congenital heart surgery does not seem to correlate with postoperative neurological injuries.

## Conclusion

Despite advances in cardiac surgery, anesthesia and CPB and despite the mortality after repair of complex congenital cardiac lesions has become rare, neurologic morbility is still significant. The incidence of neurological complications after pediatric cardiac surgery varies between 2% and 25% [28-30]. TCD technology provides the clinicians with a real time CBF evaluation. Current scientific evidence suggests a good correlation between changes in cerebral blood flow and MCA blood flow velocity. TCD can be particularly useful in assessing cerebral perfusion of children during CPB.

This technique is fast, accurate, reproducible and noninvasive. The introduction of Doppler technology has allowed an accurate monitorization of CBF during circulatory arrest and of low-flow CPB. TCD has also been utilized in detecting cerebral emboli, improper cannulation or cross clamping of aortic arch vessels. TCD plays an essential role in multimodal neurological monitorization during CPB. Limitations to TCD routine utilization are represented by the need of a learning curve and some experience by the operators. Some technical caveats must be take into consideration: if the angle of insonation is minimal and the TCD has active high-pass filter, the minimum displayed CBFV is 3–4 cm/sec, with the consistent risk of a "blind spot" in CBF detection when perfusion may be present. In conclusion, TCD represents a very important and accessible instrument able to detect an important rate of CBF modification during CPB. Nonetheless, in order to achieve a complete assessment of cerebral hemodynamics during and after heart surgery, only a multimodal approach can provide a reliable measurement of cerebral perfusion and oxygenation. Neurophysiological multimodal monitoring (NIRS, TCD, processed EEG) can help to improve neurologic outcome in a cost-effective manner.

## Abbreviations

TCD: Transcranial Doppler Ultrasound

CBF: cerebral blood flow

CBFV: cerebral blood flow velocity

CPB: cardiopulmonary bypass

MCA: middle cerebral artery

ACA: anterior cerebral artery

DHCPB: deep hypotermic cardiopulmonary bypass

DHCA: deep hypotermic cardiocirculatory arrest

CMRO_2_: cerebral metabolic rate of oxygen

CPP: cerebral perfusion pressure

RLFP: regional low flow perfusion

NIRS: near infrared spectroscopy

## Competing interests

The author(s) declare that they have no competing interests.

## Authors' contributions

AP designed the review, participated to analysed paper collection and drafted the paper.

ZR, LDC, CG, CI participated to analysed paper collection and collegial discussion.

SPS and SP revised the article.
